# Ethyl Acetate Fraction of Aqueous Extract of *Lentinula edodes* Inhibits Osteoclastogenesis by Suppressing NFATc1 Expression

**DOI:** 10.3390/ijms21041347

**Published:** 2020-02-17

**Authors:** Hyerim Lee, Kyubin Lee, Sheunghun Lee, Jisu Lee, Won Tae Jeong, Heung Bin Lim, Tae Kyung Hyun, Sun-Ju Yi, Kyunghwan Kim

**Affiliations:** 1 School of Biological Sciences, College of Natural Sciences, Chungbuk National University, Cheongju, Chungbuk 361-763, Korea; qjsro1324@gmail.com (H.L.); kblee816@hanmail.net (K.L.); lesh91@naver.com (S.L.); wltndlaos@naver.com (J.L.); sjyi@chungbuk.ac.kr (S.-J.Y.); 2 Department of Industrial Plant Science and Technology, Chungbuk National University, Cheongju 28644, Korea; shewaspretty@chungbuk.ac.kr (W.T.J.); heungbin@chungbuk.ac.kr (H.B.L.); taekyung7708@cbnu.ac.kr (T.K.H.)

**Keywords:** *Lentinula edodes*, osteoclast, NFATc1, osteoporosis

## Abstract

Bone tissue is continuously remodeled by the coordinated action of osteoclasts and osteoblasts. Nuclear factor-activated T cells c1 (NFATc1) is a well-known transcription factor for osteoclastogenesis and transcriptionally activated by the c-Fos and nuclear factor-kappa B (NF-κB) signaling pathways in response to receptor activation of NF-κB ligand (RANKL). Since excessive RANKL signaling causes an increase of osteoclast formation and bone resorption, inhibition of RANKL or its signaling pathway is an attractive therapeutic approach to the treatment of pathologic bone loss. In this study, we show that an ethyl acetate fraction (LEA) from the shiitake mushroom, *Lentinula edodes*, inhibited RANKL-induced osteoclast differentiation by blocking the NFATc1 signaling pathway. We found that the water extract and its subsequent ethyl acetate fraction of *L. edodes* significantly suppressed osteoclast formation. Comparative transcriptome analysis revealed that LEA specifically downregulated a set of RANKL target genes, including *Nfatc1*. Next, we found that LEA suppresses *Nfatc1* expression mainly through the inhibition of the transactivity of p65 and NFATc1. Moreover, treatment of LEA rescued an osteoporotic phenotype in a zebrafish model of glucocorticoid-induced osteoporosis. Collectively, our findings define an undocumented role of the shiitake mushroom extract in regulating bone development.

## 1. Introduction

Bones are constantly degraded and regenerated throughout life. This process is known as bone remodeling, which is critical for bone shape and integrity [1]. Bone remodeling is carefully regulated by osteoclasts and osteoblasts. Disturbing these processes leads to skeletal disorders, such as osteoporosis and osteopetrosis [2,3]. Glucocorticoid-induced osteoporosis (GIO) is the most frequent form of secondary osteoporosis and increases the risk of bone fractures [4]. Glucocorticoids exert adverse effects on bone metabolism, including a decrease in the number and function of osteoblasts and an increase in the life span and differentiation of osteoclasts. Long-term glucocorticoid treatment is associated with the development of GIO. 

Osteoclasts are multinucleated giant cells that are responsible for bone degradation. They are formed by the fusion of mononuclear precursors of the hematopoietic stem cell lineage [5,6]. Macrophage colony stimulating factor (M-CSF) and receptor activator of nuclear factor-kappa B ligand (RANKL) are two essential ligands for osteoclast differentiation. The binding of RANKL to the RANK receptor initiates various signaling pathways, such as NF-κB, c-JUN N-terminal kinase (JNK), p38 mitogen-activated protein kinase, and extracellular signal-related kinase pathways [7,8]. c-Fos and NF-κB signaling pathways activate nuclear factor-activated T cells c1 (NFATc1), a master transcription factor for osteoclastogenesis, subsequently stimulating the expression of diverse osteoclast-specific genes, such as tartrate resistant acid phosphatase (TRAP), cathepsin K, and osteoclast-associated receptor [9]. Since upregulated osteoclast activity is associated with osteoporosis, blocking excessive osteoclast differentiation is a potential therapeutic strategy [10,11]. In order to overcome the limitations of the currently available therapy, natural products that inhibit osteoclast differentiation are gaining interest for bone health improvement in osteoporosis [12,13].

*Lentinula edodes*, the shiitake mushroom, is the second most popular cultivated edible mushroom in the world [14]. *L. edodes* possesses antitumor activities, antioxidant activities, antiviral activities, immunomodulating properties, and antimicrobial activities [14,15,16,17,18,19,20,21]. Although the extract of *L. edodes* increased osteoblastogenesis [15,22], little is known about the effect of osteoclast differentiation of this organism. In this study, we show that *L. edodes* ethyl acetate fraction (LEA) significantly blocked osteoclast differentiation in solvent fractionation experiments. Transcriptome profiling showed that LEA negatively regulates RANKL-induced osteoclastogenesis by suppressing NFATc1 expression. Furthermore, treatment with LEA rescued the osteoporotic phenotype in an osteoporotic zebrafish model induced by prednisolone.

## 2. Results

### 2.1. LEA Suppresses Osteoclast Differentiation

**Figure 1:**
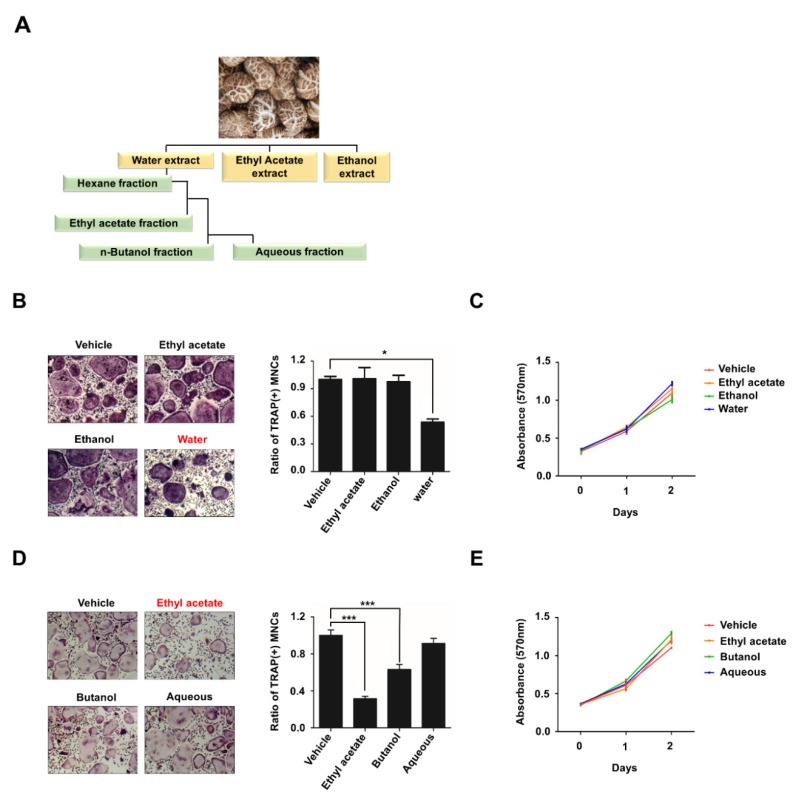
Ethyl acetate fraction of aqueous extract of *Lentinula edodes* inhibits RANKL-mediated osteoclastogenesis. (**A**) Schematic representation showing the extraction and fractionation of anti-osteoclastic compounds from *L. edodes*. (**B**) BMMs were treated with M-CSF (30 ng/mL) and RANKL (100 ng/mL) in the presence or absence of 10 μg/mL three extract (water extract, ethyl acetate extract, ethanol extract) of *L. edodes* for three days, and osteoclast formation was examined by tartrate-resistant acid phosphatase (TRAP) staining (**left**) and counting the number of TRAP-positive multinuclear osteoclasts (**right**). (**C**) BMMs were treated with M-CSF (30 ng/mL) and RANKL (100 ng/mL) in the presence of the extracts as in (**B**), and cell proliferation was measured by MTT assays. (**D**) BMMs were treated with M-CSF (30 ng/mL) and RANKL (100 ng/mL) in the presence of four fractions (10 μg/mL) of water extract of *L. edodes* for three days, and osteoclast differentiation was assessed by TRAP staining (left) and the number of TRAP-positive multinuclear osteoclasts (right) was counted. (**E**) BMMs were treated with the fractions as in (D), and cell proliferation was measured by MTT assays. Error bars represent the mean result ± SD of three independent experiments; * *p* < 0.05, *** *p* < 0.001.

To examine the effect of *L. edodes* on osteoclast differentiation, we first prepared extracts of *L. edodes* using three different solvents (Figure 1A). Then, bone marrow-derived macrophages (BMMs) as osteoclast precursors (OCP) were treated with ethyl acetate extract, ethanol extract, or water extract in the presence of M-CSF and RANKL. The formation of TRAP-positive multinucleated osteoclasts was significantly inhibited by the water extract, but not by the ethyl acetate extract or the ethanol extract (Figure 1B and Supplementary Figure S1). The findings that the water extract had no effect on the proliferation of osteoclast precursors indicate that the water extract directly controls the differentiation ability of OCP cells (Figure 1C). The water extract was further fractionated using three different organic solvents and subsequently determined for anti-osteoclastogenic activity. LEA exhibited the most potent anti-osteoclastogenic activity (Figure 1A,D). LEA rarely affected OCP proliferation (Figure 1E). 

### 2.2. LEA Modulates a Set of Osteoclast-Related Gene Expression in RANKL-Induced Osteoclastogenesis

**Figure 2:**
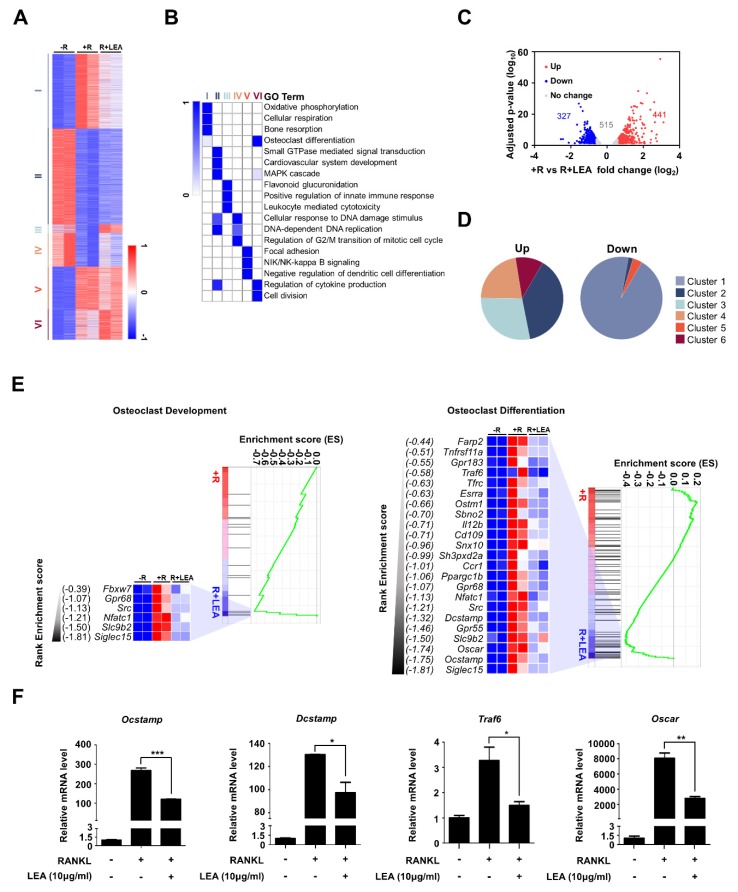
LEA alters gene expression profiling in BMMs. (**A**) K-means (K = 6) clustering of 4740 differentially expressed genes (DEGs) in any pairwise comparison among three conditions (−R; no RANKL, +R; RANKL, R + LEA; ethyl acetate fraction of *L. edodes* with RANKL). Clusters are indicated on the left. (**B**) Heatmap showing the *p*-value significance of GO term enrichment for genes in each cluster. (**C**) Volcano plot of transcriptomic changes between +R and R + LEA. Genes with increased (red) or decreased (blue) expression in LEA + RANKL-treated cells relative to RANKL-treated cells were defined based on FDR-adjusted *p* < 0.05 and greater than 1.5-fold expression changes. (**D**) Pie charts showing each cluster portion of as in (**C**). Up: Cluster 1 (0%), Cluster 2 (38.5%), Cluster 3 (28.3%), Cluster 4 (22.2%), Cluster 5(0%), and Cluster 6 (11%). Down: Cluster 1 (94.5%), Cluster 2 (1.8%), Cluster 3 (0%), Cluster 4 (0%), Cluster 5 (3.7%), and Cluster 6 (0%). (**E**) GSEA of 4740 genes as in (**A**) shows the enrichment of genes associated with osteoclast development and osteoclast differentiation. Heatmap represents core enriched genes based on their enrichment score. (**F**) qRT-PCR was performed to quantify relative mRNA levels of the representative genes for osteoclastogenesis. Error bars represent the mean result ± SD of three independent experiments; * *p* < 0.05, ** *p* < 0.01, *** *p* < 0.001.

To elucidate the inhibitory mechanism of LEA on RANKL-induced osteoclast differentiation, we performed genome-wide transcriptome analysis of BMMs with or without LEA during RANKL-mediated osteoclastogenesis. Analysis of RNA-seq data revealed 4740 differentially expressed genes in any pairwise comparison among the three conditions (no RANKL (-R), RANKL (R), RANKL + LEA (R+LEA)). K-means clustering classified the genes into six gene clusters that were differentially modulated by RANKL and LEA (Figure 2A and Supplementary Table SI). Gene ontology (GO) analysis revealed that each cluster was enriched in genes related to distinct biological functions (Figure 2B). Clusters I and VI showed the enrichment for GO terms associated with bone resorption and osteoclast differentiation. Specifically, LEA selectively downregulated cluster I, but not cluster VI (Figure 2A). Because RANKL is known to induce osteoclast-related gene expression and LEA inhibited RANKL-induced osteoclast differentiation (Figure 1D), we focused particularly on the effect of LEA on RANKL-induced genes. As shown in Figure 2C, 1283 genes were two-fold upregulated by RANKL treatment. Upon LEA treatment, a total of 768 genes (441 upregulated and 327 downregulated genes) were differentially expressed. Interestingly, most of the downregulated genes belonged to cluster I (Figure 2D and Supplementary Table SII). In addition, gene set enrichment analysis (GSEA) scoring plots showed significant enrichments of osteoclast development and osteoclast differentiation pathways (Figure 2E). Examination of the leading-edge subset of these genes identified 23 osteoclast development genes and six osteoclast differentiation genes, respectively. The datasets from GSEA were further confirmed by qRT-PCR (Figure 2F). 

### 2.3. LEA Inhibits NFATc1 Expression during Osteoclastogenesis

**Figure 3:**
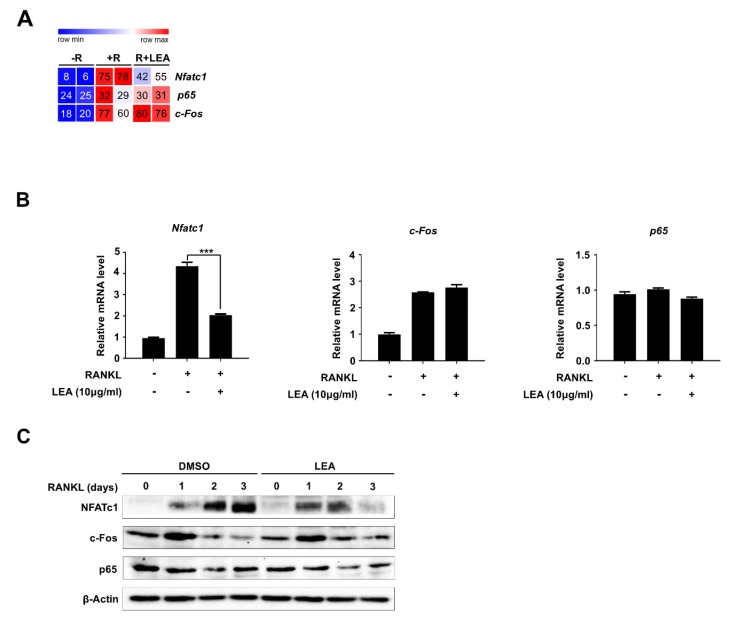
LEA represses RANKL-mediated NFATc1 expression. (**A**) Heatmap showing the reads per kilobase per million (RPKM) of c-Fos, NF-κB and NFATc1. Numbers within the figure indicate RPKM values. (**B**) Total RNA was prepared after treated with M-CSF (30 ng/mL) and RANKL (100 ng/mL) in the presence or absence of LEA (10 μg/mL), and qRT-PCR was performed using primers specific for *Nfatc1*, *c-Fos*, and *p65*. Results represents the means ± SD of three independent experiments. *** *p* < 0.001 versus only RANKL treatment. (**C**) Whole cell lysates were prepared from M-CSF/RANKL-treated BMMs with or without LEA (10 μg/mL) for 0, 1, 2, and 3 days, and analyzed by immunoblotting with NFATc1, c-Fos and p65 antibodies. β-Actin was probed as a loading control.

Given that c-Fos, NF-κB, and NFATc1 are key transcription factors involved in RANKL signaling for osteoclast differentiation [7], we first examined the effect of LEA on the mRNA expression of *c-Fos*, *p65,* and *Nfatc1*. Our RNA-seq dataset showed that LEA significantly repressed RANKL-induced *Nfatc1* expression, whereas there was no effect on *c-Fos* and *p65* expression (Figure 3A). Consistent with the RNA-seq results, our qRT-PCR analysis confirmed that LEA selectively inhibited NFATc1 expression (Figure 3B). We further evaluated the protein levels of those transcription factors. Similarly, LEA selectively diminished NFATc1 levels without affecting the protein levels of c-Fos and p65 (Figure 3C). 

### 2.4. LEA Suppresses NFATc1 Expression by Inhibiting the Transactivities of Both p65 and NFATc1

**Figure 4:**
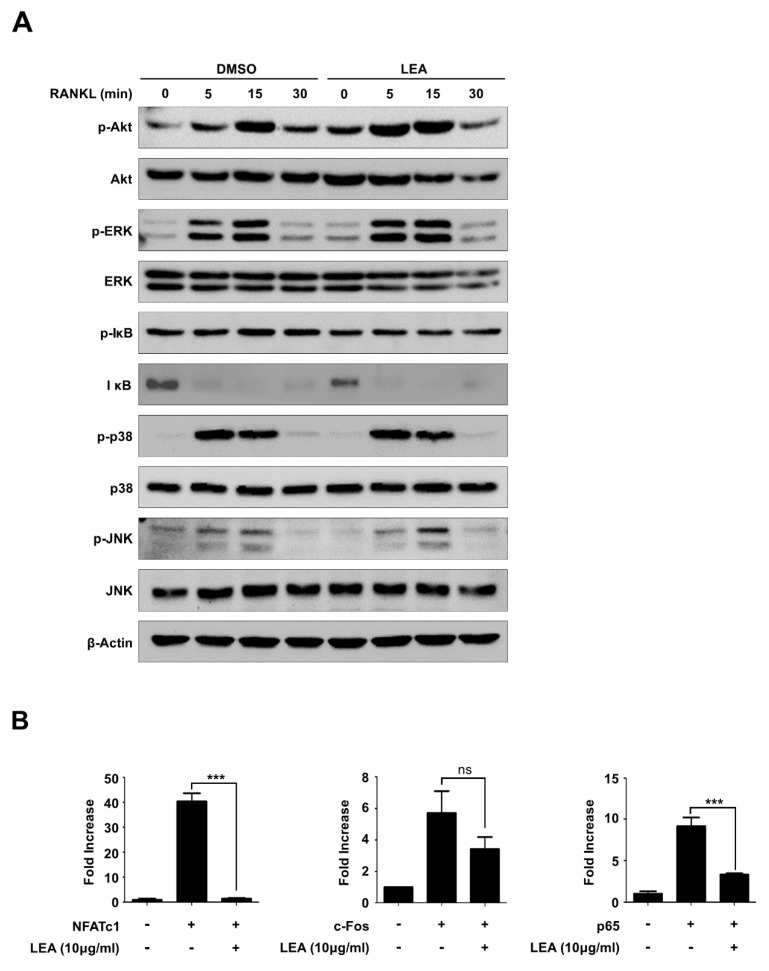
LEA suppresses NFATc1 expression by blocking both NF-κB and NFATc1-mediated transactivity. (**A**) BMMs were pre-treated with DMSO and LEA (10 μg/mL) for 1 h, and stimulated with RANKL for 0, 5, 15, and 30 min. The cells were lysed and immunoblotted with antibodies against p-Akt, Akt, p-ERK, ERK, p-IκB, IκB, p-p38, p38, p-JNK, and JNK. Band intensities were normalized to β-Actin control. (**B**) 293T cells were transiently transfected with the reporter plasmid *Nfatc1-Luc* along with *c-Fos*, *p65*, or *Nfatc1* in the presence or absence of LEA (10 μg/mL). Luciferase activity was measured after 24 h post-transfection using Luciferase assay kit (Promega, Madison, WI, USA). Each bar represents the means ± S.D. of three independent experiments. *** *p* < 0.001. ns = not significant.

It is well documented that diverse signaling pathways such as MAPKs (ERK, JNK, and p38), Akt, and NF-κB signaling pathways modulate osteoclast differentiation [23,24,25]. To gain further insight into the mechanism by which LEA controls the expression of NFATc1, we investigated whether LEA influences RANKL-induced signaling pathways, including Akt, ERK, p38, JNK, and NF-κB. BMMs cultured with M-CSF for 24 h were pretreated with DMSO or LEA for 1 h and subsequently stimulated with RANKL for the indicated times (Figure 4A). We observed that RANKL treatment increased the activation of Akt, ERK, NF-κB, p38, and JNK within 15 min. However, LEA treatment had no obvious changes in RANKL-induced signaling pathways (Figure 4A). 

Because c-Fos and NF-κB, as well as NFATc1 itself, transactivate *Nfatc1* expression, we performed an *Nfatc1* luciferase reporter gene assay to determine whether LEA affects their transactivities. Expression of NFATc1, c-Fos, or p65 increased *Nfatc1* reporter gene transcription. LEA treatment almost completely abrogated transactivities of NFATc1 and p65, but has a modest inhibitory effect on c-Fos transactivity (Figure 4B).

### 2.5. LEA Suppresses Prednisolone-Induced Osteoporosis in Zebrafish Larvae

To examine the in vivo efficacy of LEA against osteoporosis, we employed zebrafish for this study since zebrafish are an ideal model system for the in vivo analysis of GIO [26,27].

**Figure 5:**
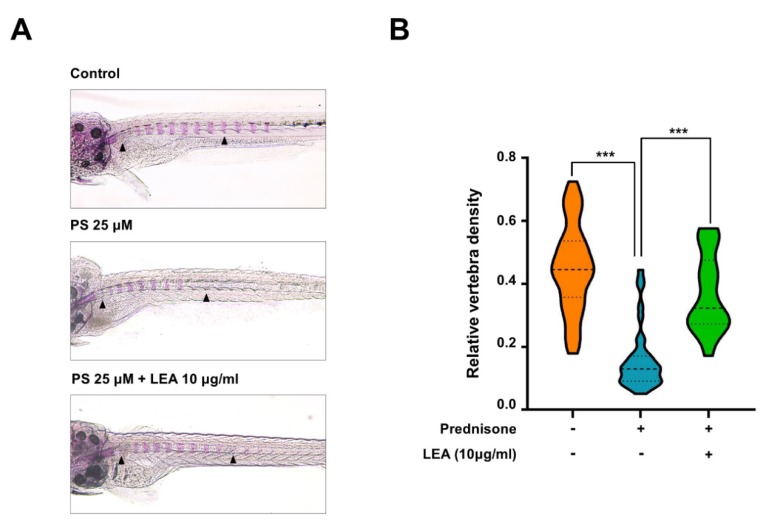
Anti-osteoporotic effect of LEA in a model of prednisolone-induced osteoporosis in zebrafish. (**A**) The larvae at 10 dpf (days post-fertilization) were treated with 25 µM prednisolone (PS) in the presence or absence of LEA (10 μg/mL) for 3 days. Whole-mount Alizarin red staining was performed to analyze the mineralized bone. Arrowheads indicate the bone Alizarin red staining. (**B**) Relative vertebral bone density was assessed by measuring the areas of the first ten stained vertebrae (V1–V10, indicated by arrowhead in (A)). *** *p* < 0.001 versus only prednisolone treatment.

Zebrafish larvae at 10 days post-fertilization were treated with or without LEA in the presence of prednisolone (25 μM) for three days, then whole-mount bone staining was performed. We observed that bone mineralization was severely decreased by prednisolone. However, LEA treatment reduced prednisolone-induced osteoporosis (Figure 5).

## 3. Discussion

Although the shiitake mushroom possesses diverse bioactivities, such as immune modulation, antitumor, liver protection, cholesterol lowering, antiviral, and blood pressure lowering activities, there are few reports describing the effects of *L. edodes* on osteoblastogenesis or osteoclastogenesis [26,27]. Saif et al. demonstrated that the *L. edodes* water extract showed in vitro bone-inducing effects on human osteoblastic cells [22]. Another study demonstrated that water extracts of mushrooms decrease bone resorption and improve bone formation in vivo [28]. However, the effects of *L. edodes* extracts on osteoclastogenesis have yet to be fully elucidated. In the present study, we investigated the potential role of the shiitake mushroom in modulating the differentiation process in which osteoclast precursors are differentiated into mature osteoclast by RANKL. Using solvent fractionation methods, we discovered that the ethyl acetate fraction of aqueous extracts of *L. edodes* had strong anti-osteoclastogenic activity. Recently, Fang et al. showed that an ethyl acetate fraction of aqueous methanol extracts of shiitake mushroom induced apoptosis in cancer cells by arresting cell cycle [15]. It will be interesting to examine the effects of the LEA used in this study on other biological activities.

Our transcriptome analysis demonstrated that LEA negatively regulated a set of osteoclast-related gene expression. It is noteworthy that LEA significantly inhibited RANKL-induced NFATc1 expression, which is a key transcription factor for osteoclastogenesis. Additionally, GSEA analysis suggested that LEA treatment controlled expressions of *Tnfrsf11a/Rank*, which is a key regulator of osteoclastogenesis via RANKL-RANK signaling (Figure 2E) [29]. DEG analysis, however, showed that *Rank* gene is not significant with a cutoff of FDR-adjusted *p* < 0.05 and 1.5-fold (Figure 2C and Supplementary Table SII). Moreover, our qRT-PCR study clearly revealed that LEA treatment had no apparent effect on MCSF- or RANKL-mediated *Rank* expression (Supplementary Figure S2). These results indicate that LEA may suppress *Nfatc1* expression by regulating RANK downstream rather than *Rank* expression. 

NFATc1 expression during osteoclastogenesis is tightly regulated via the NF-κB and c-Fos signaling pathways [7,30]. The NF-κB components p65 and p50 are recruited to *Nfatc1* promoter upon RANKL stimulation. Calcium signal-induced activation of NFATc1 also triggers *Nfatc1* expression by binding to its own promoter in cooperation with c-Fos [31]. In our study, LEA did not affect cellular signaling of NF-κB and c-Fos activation, as well as their transcription and translation. Interestingly, LEA selectively abolished NF-κB/NFATc1 transactivation for NFATc1 expression and did partially affect c-Fos transactivation. These results suggest that LEA might block p65 and NFATc1 localization at the *Nfatc1* promoter. Similarly, Ha et al. observed that water extract of Uncaria sinensis inhibits RANKL-induced NF-κB transactivation without modulating the activation of MAPK and NF-κB by RANKL [32]. Additional studies are needed to understand the exact mechanism underlying the effect of LEA on NFATc1 expression.

Since LEA efficiently suppressed osteoclast differentiation in this study, we anticipate that LEA will be effective for osteoporosis treatment. GIO is one of the serious side effects of glucocorticoid treatment, resulting in vertebra fractures. Recent studies revealed that glucocorticoids produce osteoporosis simultaneously by stimulating osteoclastogenesis and attenuating osteoblast formation [2,4]. Furthermore, we and others have shown that prednisolone treatment induces an osteoporosis phenotype in zebrafish larvae [26,27]. Based on our findings that LEA treatment prevented bone loss in a zebrafish GIO model, we believe that LEA prevents glucocorticoid-induced bone loss by affecting both osteoclasts and osteoblasts. Further studies are required to isolate bioactive compounds from LEA. Nevertheless, our present results indicate that LEA could have therapeutic value in treating GIO.

## 4. Materials and Methods

### 4.1. Preparation of Water Extract and Its Fractions Using L. edodes

Fresh fruiting bodies of *L. edodes* strain Chamaram were sliced and freeze-dried for 72 h. 150 g of the freeze-dried materials were soaked in various solvents (water, ethyl acetate, or ethanol) for 6 h and sonicated for 1 h in an ultrasonic bath (DAIHAN-Sci, Seoul, Korea). After filtration, each extract was evaporated using a rotary vacuum evaporator (RV8, Germany). The solvent fractions of crude water extract have been prepared as previously described with minor modifications [33]. The crude water extract (59.5 g) was suspended in water, and then sequentially partitioned with equal volume of hexane, ethyl acetate (EtOAc) and n-butanol (BuOH). The remaining aqueous extract was used as an aqueous fraction. Each fraction was evaporated using vacuum evaporator. The yields afforded EtOAc (1.6% *w/w*), BuOH (7.7% *w/w*) and aqueous (90.7% *w/w*) fractions. The hexane fraction exhibited the lowest yield of 0.01%, thus, this fraction was excluded from the next analysis.

### 4.2. Osteoclast Differentiation and TRAP Staining

Osteoclast precursor cells were prepared as previously described [34]. In brief, bone marrow cells were collected by flushing tibias and femurs from six- to eight week-old ICR male mice. Cells were cultured in α-minimum essential medium supplemented with 10% FBS and M-CSF (5 ng/mL) for 16 h. Non-adherent cells were harvested and cultured with M-CSF (30 ng/mL) for three days. Suspended cells were removed and adherent cells were used as OCP cells. Osteoclast differentiation were performed as recently described [35]. Briefly, OCP cells were cultured in 48-well plates with 30 ng/mL of M-CSF and 100 ng/mL of RANKL, in the presence or absence of LEA (10 μg/mL). On day 3, the cells were fixed with 3.7% formaldehyde in phosphate-buffered saline and stained for TRAP using an acid phosphatase leukocyte kit (Sigma-Aldrich, St. Louise, MO, USA). TRAP-positive, multinucleated cells containing three or more nuclei were counted as osteoclasts under a light microscope. 

### 4.3. Cell Viability Assay

To examine the effect of *L. edodes* on cell proliferation, osteoclast precursor cells were treated with the extracts of the *L. edodes* (EtOAc, ethanol, and water) or the fractions of the *L. edodes* (EtOAc, BuOH, and aqueous), and MTT assays were performed after 24 or 48 h using Cell Proliferation KIT 1 (Roche Diagnostics, Mannheim, Germany).

### 4.4. RNA-seq

Total RNA was isolated using RNeasy Mini kit (Qiagen, Hilden, Germany). Libraries were prepared from 2 μg of total RNA using the SMARTer Stranded RNA-Seq Kit (Clontech Laboratories, Inc., Palo Alto, Santa Clara, CA, USA). High-throughput sequencing was performed as paired-end 100 sequencing using HiSeq 2500 (Illumina, Inc., San Diego, CA, USA). mRNA-Seq reads were mapped to reference mouse genome (mm10 assembly) using HISAT2 with default parameters. RNA expression was quantified using the analyzeRepeats.pl command in HOMER. DEGs were assessed with DESeq2 using getDiffExpression.pl in HOMER. The Cut-offs parameters for significantly up- or down-regulated genes were set at fold change  ≥1.5 and FDR < 0.05 (Benjamin-Hochberg). To generate the heatmap of K-means clustering, we used the Morpheus web site (https://software.boradinstitute.org/morpheus/). To find GO terms enriched in DEGs, we performed Metascape tool [36]. Gene Set Enrichment Analysis were performed on the entire genes (a total of 24940 genes) using MsigDB gene sets [37].

### 4.5. RT-qPCR

Total RNA was isolated from osteoclasts and reverse-transcribed using the Moloney Murine Leukemia Virus(M-MLV) reverse transcriptase (Promega, Madison, WI, USA). Real-time PCR was performed using IQ SYBR Green SuperMix (Bio-Rad, Hercules, CA, USA). The sequences of primers used for qPCR were as follows: *Nfatc1* 5′-CTCGAAAGACAGCACTGGAGCAT-3′ (forward) and 5′-CGGCTGCCTTCCGTCTCATAG-3′ (reverse); *p65* 5′-GGAGTTCCAGTACTTGCC-3′ (forward) and 5′-GTCCTTTTGCGCTTCTCT-3′ (reverse); *c-Fos* 5′-CCAGTCAAGAGCATCAGCAA-3′ (forward) and 5′-AAGTAGTCGCAGCCCCGAGTA-3′ (reverse); *DC-stamp* 5′-CCGCTGTGGACTATCTGCTG-3′ (forward) and 5′-CTCAATGGCTGCTTTGATCG-3′ (reverse); *OC-stamp* 5′-CTGTGGTGCCAAACGTCTTA-3′ (forward) and 5′-TCTCCTGAGTGATCGTGTGC-3′ (reverse); *Traf6* 5′-AAACCACGAAGAGGTCATGG-3′ (forward) and 5′-GCGGGTAGAGACTTCACAGC-3′ (reverse); *Oscar* 5′-CTGCTGGTAACGGATCAGCTCCCCAGA-3′ (forward) and 5′-CCAAGGAGCCAGAACCTTCGAAACT-3′ (reverse); *Rank* 5′- GCTGGCTACCACTGGAACTC-3′ (forward) and 5′-GTGCAGTTGGTCCAAGGTTT (reverse).

### 4.6. Western Blot Analysis

Whole cell lysates were prepared with lysis buffer (25 mM Tris, pH 7.9, 150 mM NaCl, 0.5% NP-40, 1 mM EDTA, 5% glycerol, protease inhibitor cocktails, 1 mM sodium orthovanadate, and 2.5 mM sodium pyrophosphate). Protein concentration of cell lysates was determined by the Protein Assay Kit (iNtRON biotechnology, Inc., Seoul, Korea). Cell lysates were separated by SDS-PAGE and transferred to PVDF membranes (GE Healthcare, Freiburg, Germany), and subject to Western blotting using the indicated antibodies. The antibodies used in this study were as follows: p-Akt, Akt, p-ERK and ERK antibodies from Cell Signaling Technology (Danvers, MA, USA); p-IκB, NFATc1 and p65 antibodies from Santa Cruz (Santa Cruz, CA, USA); IκB and β-Actin antibodies from Sino Biological (Beijing, China); and p-p38, p38, p-JNK, JNK, and c-Fos antibodies from Millipore (Burlington, MA, USA).

### 4.7. Reporter Gene Assay

293T cells were plated in 12-well plates at 80% confluence and transfected with an *Nfatc1-Luc* reporter plasmid and expression vectors for *c-Fos*, *p65*, and *Nfatc1* in the presence or absence of LEA (10 μg/mL) for 24 h. Cells were lysed in Reporter Lysis buffer (Promega, Madison, WI, USA) and assayed for luciferase activity using SpectraMax i3x (Molecular Devices, San Jose, CA, USA). 

### 4.8. Fish Maintenance and Drug Treatment

Zebrafish were raised under standard maintaining conditions in a circulating water system at 28 °C, with day–night (14 h light/10 h dark) cycles. The fish were fed live brine shrimp three times a day. The male and female zebrafish with high potential to produce fertilized eggs were chosen for spawning. After removing the unfertilized eggs, embryos were kept at 28 °C, with day–night (14 h light/10 h dark) cycles. At 10 dpf, the larvae were treated with 25 µM prednisolone of LEA (10 μg/mL). At 13 dpf, the larvae were collected for whole-mount skeletal staining. All experimental protocols were approved by the Animal Care and Use Committee of the Chungbuk National University (CBNUA-1244-19-02, 8 March 2019).

### 4.9. Whole-Mount Skeletal Staining

Alizarin red staining on zebrafish larvae was performed as previously described [35]. Briefly, larvae at 13 dpf were fixed in 10% neutral buffered formalin. After bleaching pigmentation with 3% H_2_O_2_ solution, the larvae were stained with 1 mg/mL alizarin red stain/1% KOH, pH 4.2 and then sequentially washed with 20% glycerol/1% KOH, 40% glycerol/1% KOH, and 60% glycerol/1% KOH. Images of stained larvae were obtained using a stereo microscope SMZ18 (Nikon, Tokyo, Japan). For quantification of bone mineral density, the areas of the first ten stained vertebrae (V1–V10) were calculated using the ImageJ densitometry program.

### 4.10. Statistical Analysis

All quantitative data were presented as mean ± SD. GraphPad Prism (GraphPad Software Inc. La Jolla, CA, USA) was used for all analyses. Statistical analysis was done using the one-way ANOVA test, followed by Tukey’s Multiple Comparison Test.

### 4.11. Accession Numbers

RNA-seq datasets were deposited at NCBI Gene Expression Omnibus (GSE 142866).

## Supplementary Materials

Supplementary materials can be found at https://www.mdpi.com/1422-0067/21/4/1347/s1.

## Abbreviations

RANKL	Receptor activator of the NF-κB ligand
BMM	Bone marrow-derived macrophage
GIO	Glucocorticoid-induced osteoporosis
TRAP	Tartrate resistant acid phosphatase

## References

[1] Florencio-Silva R., Sasso G.R., Sasso-Cerri E., Simoes M.J., Cerri P.S. (2015). Biology of Bone Tissue: Structure, Function, and Factors That Influence Bone Cells. BioMed Res. Int..

[2] Crockett J.C., Rogers M.J., Coxon F.P., Hocking L.J., Helfrich M.H. (2011). Bone remodelling at a glance. J. Cell Sci..

[3] Sobacchi C., Schulz A., Coxon F.P., Villa A., Helfrich M.H. (2013). Osteopetrosis: Genetics, treatment and new insights into osteoclast function. Nat. Rev. Endocrinol..

[4] Canalis E., Giustina A., Bilezikian J.P. (2007). Mechanisms of anabolic therapies for osteoporosis. N. Engl. J. Med..

[5] Charles J.F., Aliprantis A.O. (2014). Osteoclasts: More than ‘bone eaters’. Trends Mol. Med..

[6] Yi S.J., Lee H., Lee J., Lee K., Kim J., Kim Y., Park J.I., Kim K. (2019). Bone Remodeling: Histone Modifications as Fate Determinants of Bone Cell Differentiation. Int. J. Mol. Sci..

[7] Kim J.H., Kim N. (2014). Regulation of NFATc1 in Osteoclast Differentiation. J. Bone Metab..

[8] Lee N.K., Choi Y.G., Baik J.Y., Han S.Y., Jeong D.W., Bae Y.S., Kim N., Lee S.Y. (2005). A crucial role for reactive oxygen species in RANKL-induced osteoclast differentiation. Blood.

[9] Boyce B.F., Yamashita T., Yao Z., Zhang Q., Li F., Xing L. (2005). Roles for NF-kappaB and c-Fos in osteoclasts. J. Bone Miner. Metab..

[10] Cherian K.E., Kapoor N., Paul T.V. (2017). Glucocorticoid-induced Osteoporosis. Indian J. Endocrinol. Metab..

[11] Rodan G.A., Fleisch H.A. (1996). Bisphosphonates: Mechanisms of action. J. Clin. Investig..

[12] Cheon Y.H., Baek J.M., Park S.H., Ahn S.J., Lee M.S., Oh J., Kim J.Y. (2015). *Stauntonia hexaphylla* (*Lardizabalaceae*) leaf methanol extract inhibits osteoclastogenesis and bone resorption activity via proteasome-mediated degradation of c-Fos protein and suppression of NFATc1 expression. BMC Complement. Altern. Med..

[13] Hwang Y.H., Jang S.A., Kim T., Ha H. (2019). Forsythia suspensa Protects against Bone Loss in Ovariectomized Mice. Nutrients.

[14] Bisen P.S., Baghel R.K., Sanodiya B.S., Thakur G.S., Prasad G.B. (2010). *Lentinus edodes*: A macrofungus with pharmacological activities. Curr. Med. Chem..

[15] Fang N., Li Q., Yu S., Zhang J., He L., Ronis M.J., Badger T.M. (2006). Inhibition of growth and induction of apoptosis in human cancer cell lines by an ethyl acetate fraction from shiitake mushrooms. J. Altern. Complement. Med..

[16] Finimundy T.C., Gambato G., Fontana R., Camassola M., Salvador M., Moura S., Hess J., Henriques J.A., Dillon A.J., Roesch-Ely M. (2013). Aqueous extracts of *Lentinula edodes* and *Pleurotus sajor-caju* exhibit high antioxidant capability and promising in vitro antitumor activity. Nutr. Res..

[17] Gu Y.H., Belury M.A. (2005). Selective induction of apoptosis in murine skin carcinoma cells (CH72) by an ethanol extract of *Lentinula edodes*. Cancer Lett..

[18] Hirasawa M., Shouji N., Neta T., Fukushima K., Takada K. (1999). Three kinds of antibacterial substances from *Lentinus edodes* (Berk.) Sing. (Shiitake, an edible mushroom). Int. J. Antimicrob. Agents.

[19] Huang W., Kim J.S., Chung H.Y. (2011). Antioxidant activity and total phenolic content in shiitake mycelial exudates. Nat. Prod. Commun..

[20] Lull C., Wichers H.J., Savelkoul H.F. (2005). Antiinflammatory and immunomodulating properties of fungal metabolites. Mediat. Inflamm..

[21] Tanaka K., Matsui Y., Ishikawa S., Kawanishi T., Harada M. (2012). Oral ingestion of Lentinula edodes mycelia extract can restore the antitumor T cell response of mice inoculated with colon-26 cells into the subserosal space of the cecum. Oncol. Rep..

[22] Saif A., Wende K., Lindequist U. (2013). In vitro bone inducing effects of *Lentinula edodes* (shiitake) water extract on human osteoblastic cell cultures. Nat. Prod. Bioprospecting.

[23] Negishi-Koga T., Takayanagi H. (2009). Ca2+-NFATc1 signaling is an essential axis of osteoclast differentiation. Immunol. Rev..

[24] Ross F.P. (2006). M-CSF, c-Fms, and signaling in osteoclasts and their precursors. Ann. N. Y. Acad. Sci..

[25] Wada T., Nakashima T., Hiroshi N., Penninger J.M. (2006). RANKL-RANK signaling in osteoclastogenesis and bone disease. Trends Mol. Med..

[26] Barrett R., Chappell C., Quick M., Fleming A. (2006). A rapid, high content, in vivo model of glucocorticoid-induced osteoporosis. Biotechnol. J..

[27] He H., Wang C., Tang Q., Yang F., Xu Y. (2018). Possible mechanisms of prednisolone-induced osteoporosis in zebrafish larva. Biomed. Pharmacother. Biomed. Pharmacother..

[28] Erjavec I., Brkljacic J., Vukicevic S., Jakopovic B., Jakopovich I. (2016). Mushroom Extracts Decrease Bone Resorption and Improve Bone Formation. Int. J. Med. Mushrooms.

[29] Jones D.H., Kong Y.Y., Penninger J.M. (2002). Role of RANKL and RANK in bone loss and arthritis. Ann. Rheum. Dis..

[30] Takayanagi H., Kim S., Koga T., Nishina H., Isshiki M., Yoshida H., Saiura A., Isobe M., Yokochi T., Inoue J. (2002). Induction and activation of the transcription factor NFATc1 (NFAT2) integrate RANKL signaling in terminal differentiation of osteoclasts. Dev. Cell.

[31] Asagiri M., Sato K., Usami T., Ochi S., Nishina H., Yoshida H., Morita I., Wagner E.F., Mak T.W., Serfling E. (2005). Autoamplification of NFATc1 expression determines its essential role in bone homeostasis. J. Exp. Med..

[32] Ha H., Shim K.S., Ma J.Y. (2017). Water extract of *Uncaria sinensis* suppresses RANKL-induced bone loss by attenuating osteoclast differentiation and bone resorption. Integr. Med. Res..

[33] Geidam Y.A., Ambali A.G., Onyeyili P.A. (2007). Phytochemical Screening and Antibacterial Properties of Organic Solvent Fractions of *Psidium guajava* Aqueous Leaf Extracts. Int. J. Pharmacol..

[34] An D., Kim K., Lu W. (2014). Defective entry into mitosis 1 (Dim1) negatively regulates osteoclastogenesis by inhibiting the expression of nuclear factor of activated T-cells, cytoplasmic, calcineurin-dependent 1 (NFATc1). J. Biol. Chem..

[35] Kim Y., Kim J., Lee H., Shin W.R., Lee S., Lee J., Park J.I., Jhun B.H., Kim Y.H., Yi S.J. (2019). Tetracycline Analogs Inhibit Osteoclast Differentiation by Suppressing MMP-9-Mediated Histone H3 Cleavage. Int. J. Mol. Sci..

[36] Zhou Y., Zhou B., Pache L., Chang M., Khodabakhshi A.H., Tanaseichuk O., Benner C., Chanda S.K. (2019). Metascape provides a biologist-oriented resource for the analysis of systems-level datasets. Nat. Commun..

[37] Subramanian A., Tamayo P., Mootha V.K., Mukherjee S., Ebert B.L., Gillette M.A., Paulovich A., Pomeroy S.L., Golub T.R., Lander E.S. (2005). Gene set enrichment analysis: A knowledge-based approach for interpreting genome-wide expression profiles. Proc. Natl. Acad. Sci. USA.

