# Supplementary material for: Ethyl Acetate Fraction of Aqueous Extract of Lentinula edodes Inhibits Osteoclastogenesis by Suppressing NFATc1 Expression
Source: Int J Mol Sci. 2020 Feb 17;21(4):1347. doi: 10.3390/ijms21041347 (PMC7072883; doi:10.3390/ijms21041347)
Supplement: Supplementary file 1 [file ijms-21-01347-s001.zip › Supplementary Figures.docx]

**Ethyl acetate fraction of aqueous extract of *Lentinula edodes* inhibits osteoclastogenesis by suppressing NFATc1 expression**

Hyerim Lee, Kyubin Lee, Sheunghun Lee, Jisu Lee, Won Tae Jeong, Heung Bin Lim, Tae Kyung Hyun, Sun-Ju Yi, and Kyunghwan Kim


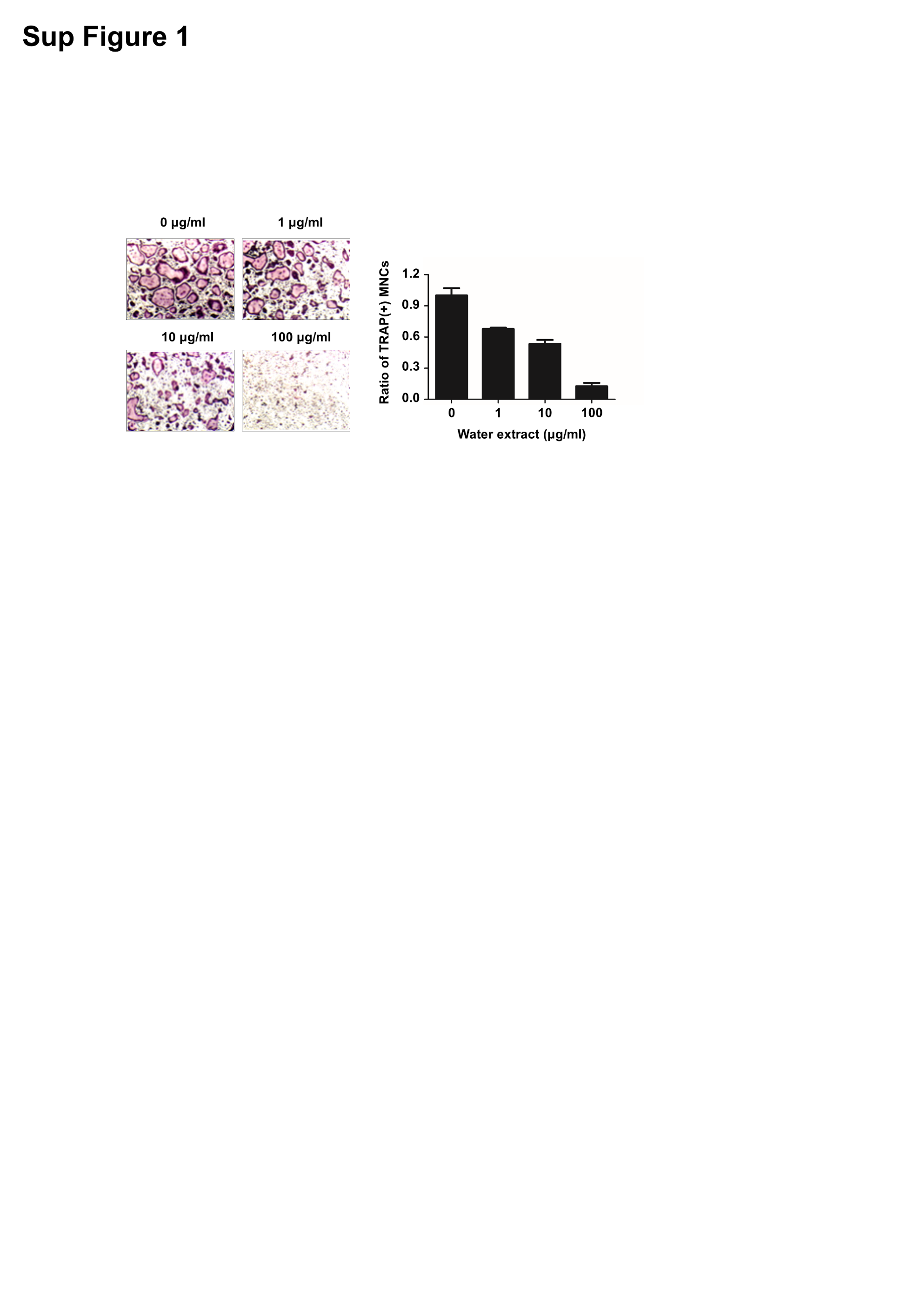


**Figure S1.**Inhibitory effect of water extract of *L. edodes* on osteoclastogensis.


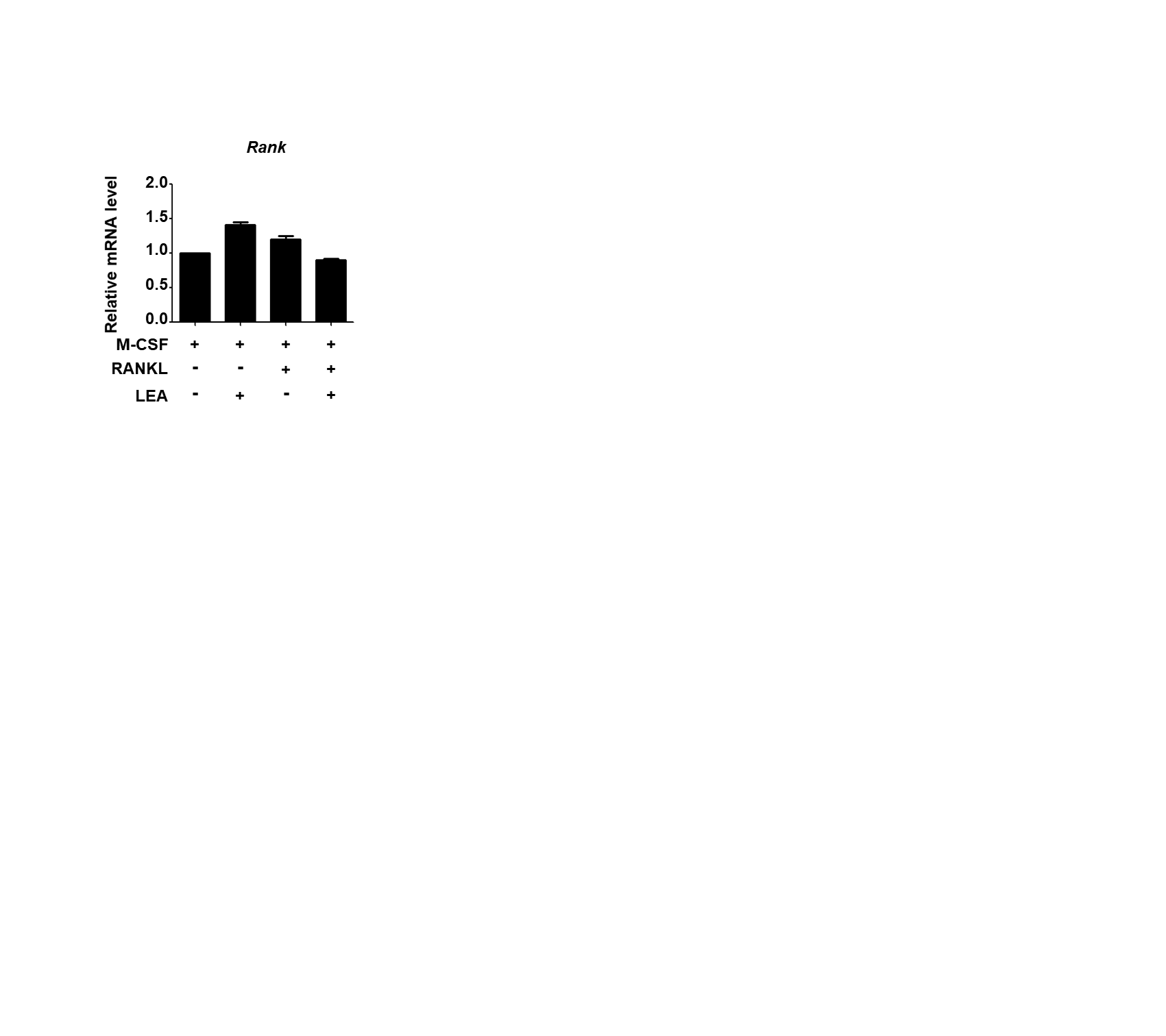


**Figure S2.  E**ffect of LEA on M-CSF- or RANKL-mediated RANK expression.
